# Detection of Cytotoxic Activity of Lectin on Human Colon Adenocarcinoma (Sw480) and Epithelial Cervical Carcinoma (C33-A)

**DOI:** 10.3390/molecules16032107

**Published:** 2011-03-02

**Authors:** Carmen Valadez-Vega, Gerardo Alvarez-Manilla, Leticia Riverón-Negrete, Alejandro García-Carrancá, José A. Morales-González, Clara Zuñiga-Pérez, Eduardo Madrigal-Santillán, Jaime Esquivel-Soto, Cesar Esquivel-Chirino, Roberto Villagómez-Ibarra, Mirandeli Bautista, Ángel Morales-González

**Affiliations:** 1 Instituto de Ciencias de la Salud, Universidad Autónoma del Estado de Hidalgo, Ex-Hacienda de la Concepción, Tilcuautla, 42080 Pachuca de Soto, Hgo, Mexico; E-Mails: jmorales101@yahoo.com.mx (J.A.M-G); zupecl@yahoo.com.mx (C.Z.-P.); eomsmx@yahoo.com.mx (E.M.-S.); mirandeli@hotmail.com (M.B.); 2 Ezose Sciences Inc, Pine Brook, New Jersey 07058, USA; E-Mail: gmanilla@ezose.com; 3 Parasitología Experimental, Instituto Nacional de Pediatría, Mexico, D.F., Mexico; E-Mail: riveron15@gmail.com; 4 Laboratorio de Virus y Cáncer, Unidad de Investigación Biomédica en Cáncer. Instituto de Investigaciones Biomédicas, Universidad Nacional Autónoma de México and Instituto Nacional de Cancerología, Secretaría de Salud, Mexico, D.F., Mexico; E-Mail: carranca@correo.biomedicas.unam.mx; 5 Facultad de Odontología, Universidad Nacional Autónoma de México (UNAM), Mexico, D.F., Mexico; E-Mails: jaime_esquivel2003@hotmail.com (J.E.-S.); cesquivelch@gmail.com (C.E.-C.); 6 Instituto de Ciencias Básicas e Ingeniería, Universidad Autónoma del Estado de Hidalgo, Pachuca, Hidalgo, Mexico; E-Mail: Roberto_ibarrav@hotmail.com; 7 Escuela Superior de Cómputo, Instituto Politécnico Nacional, México, D.F., Mexico; E-Mail: anmorales@ipn.mx

**Keywords:** lectins, tepary beans, cytotoxicity, tepary bean lectins

## Abstract

Lectins comprise a heterogeneous class of proteins that recognize the carbohydrate moieties of glycoconjugates with high specificity. Numerous studies have shown that lectins are capable of recognizing specific carbohydrate moieties displayed by malignant cells or tissues. The present work was performed to investigate the effects of tepary bean (*Phaseolus acutifolius*) lectins on proliferation, colony formation, and alteration of DNA synthesis of human malignant cells. Tepary bean lectin showed dose dependent effects on the inhibition of viability as well as on colony formation in two human malignant cells lines (C33-A, Sw480); By contrast, tepary bean lectin only showed significant effects on DNA synthesis on Sw480 cells. Our results provide evidence of the anti- proliferative and cytotoxic effects of the tepary bean lectins on C33-A and Sw480 cells lines.

## 1. Introduction

Cancer represents the principal cause of death worldwide [1,2,3]. One strategy for cancer prevention is chemoprevention, which refers to the use of natural or synthetic substances to reduce the risks of developing cancer or to reduce the probability that cancer will recur. Chemoprevention research efforts started in the early 1980s and have grown considerably since that time [4,5].

The cancer inhibitory action of natural products derived from plants has been confirmed in different animal tumor models [6,7] and has led to an increased emphasis on cancer prevention strategies in which these dietary factors may be utilized. Previous studies have demonstrated that natural agents can inhibit the development of human tumor cells *in vitro* [8,9,10].

Lectins are widely distributed in Nature, mainly in the plant kingdom, although they also occur in other organisms, such as animals and microorganisms [11,12,13,14]. Lectins are proteins or glycoproteins capable of agglutinating blood cells (erythrocytes or leukocytes) by interacting with specific carbohydrate residues in their cell membrane structure [15,16]. Although lectins have been known for more than a century, they became a focus of interest when it was found that they interact with specific carbohydrate residues on the cell membrane [15,16]. Given their specificity, lectins have the capability of distinguishing between different cell types, such as normal and malignant cells [17,18,19].

Many specific alterations in the structure of carbohydrates in the cell membrane have been observed in cancer cells, such as an increase in sialylation and modifications in the branching of complex carbohydrates; and even occasionally, the emergence of unusual structures [15,20]. For this reason, lectins represent a potentially useful tool to recognize specific alterations in transformed cells [14]. Aberrant glycosylation, which can be detected by lectin histochemistry, may predict the outcome of some tumor entities, such as in the case of aviscumine (recombinant mistletoe lectin) [21] and phytohaemagglutinin-L (PHA-L), which in addition, possess cytotoxic effects on malignant cells, and therefore may also be used in anti-tumor therapy [22].

Dietary lectins, such as wheat germ agglutinin (WGA), can inhibit cell growth of human breast cancer cells lines and may induce apoptosis after 30 min of incubation with tumor cells (Jurkat-R cells) [23,24]. Lectins from *Solanum tuberosum, Triticum vulgaris, Lycopersicon esculentum* and *Helix pomatia* has shown significant cytotoxic effects on transformed cell lines from conjunctive and corneal tissues [25]. Mistletoe lectins I,-II and III are highly toxic to several normal and malignant cells lines, thus inhibiting their proliferation [26]. Schwartz *et al.* [27] reported that concanavalin A (Con A), phytohaemagglutinin-L (PHA-L) and WGA inhibit DNA biosynthesis in pancreatic cancer cells. Other reports indicate that *Griffonia simplicifolia* agglutinin (GSA), Con A, and PHA-L had differential effect on cell growth on human colorectal cancer cell lines, by inhibiting their growth [28]. Plant lectins have also shown potential use in other diseases; for example, *Galanthus nivalis* lectin has shown inhibitory activity towards human immunodeficiency virus type I [29]; and *Maackia amurensis*, as well as *Sambucus nigra* agglutinins, have been shown to recognize specific receptors on influenza A viruses [30]. The aim of this study was investigate the cytotoxic effect of tepary bean (*Phaseolus acutifolius*) lectins (TBL) on human malignant cell lines using different *in vitro* proliferation and cytotoxic assays.

## 2. Results and Discussion

### 2.1. Tepary bean lectin purification

Lectin purification by fetuin-agarose affinity column chromatography showed that the lectin was recovered in one peak in fraction 34 when the column was washed with a solution of glycine-HCl (50 mM), pH 2.8. The hemagglutination assays employing human erythrocytes blood types A and O showed a high biological activity (Table 1); however only 10% of the hemagglutination activity measured in the crude extract was recovered. These results suggest that there are other fractions with hemagglutinating activity that did not bind to the fetuin affinity column. The purified fraction was employed for biological assays.

**Table 1:** Hemagglutination activity of the pure lectin from *Phaseolus acutifolius*.

Lectin	Lectin Concentration (mg/mL)	Human erythrocytes	
		Type A	Type O
Tepary	3.5	292.6 ± 32.9	36.6 ± 4.3

**Figure 1:**
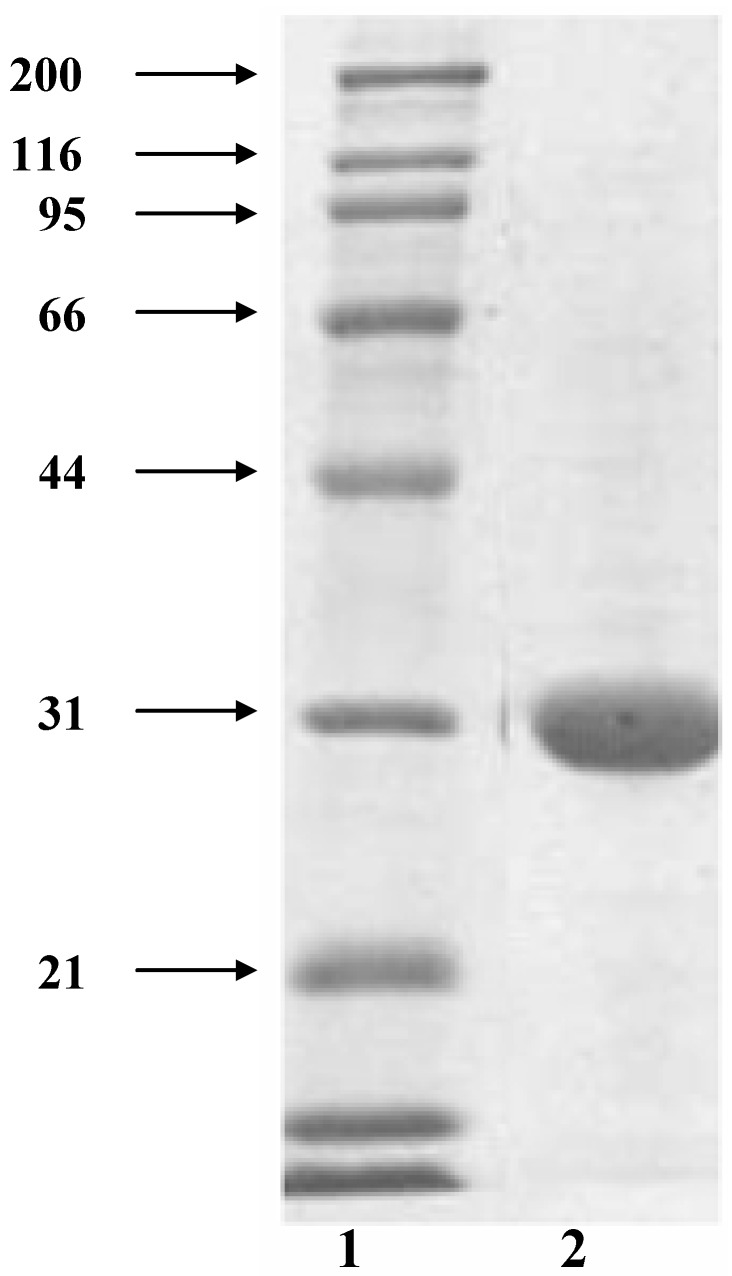
SDS-poliacrilamide electrophoresis pattern of the purified tepary bean (*Phaseolus acutifolius*) lectin. Lane 2, purified tepary bean lectin from fetuin affinity chromatography. Lane 1, molecular weight markers: myosin (200 kDa), b-galactosidase (116 kDa), phosphorylase (97 kDa), bovine serum albumin (66 kDa), carbonic anhydrase (31 kDa), trypsin inhibitor from glycine max (21 kDa).

Figure 1 shows the electrophoretic pattern on the reducing SDS-PAGE of the purified lectin showing a single band indicating the protein was purified, with a molecular weight of 31 kDa. The Native-PAGE showed a band with a molecular weight range between 132 to 153 kDa. This result suggests that the purified lectin is formed by four subunits.

### 2.2. Effect of the TBL on the viability of human malignant cells

**Figure 2:**
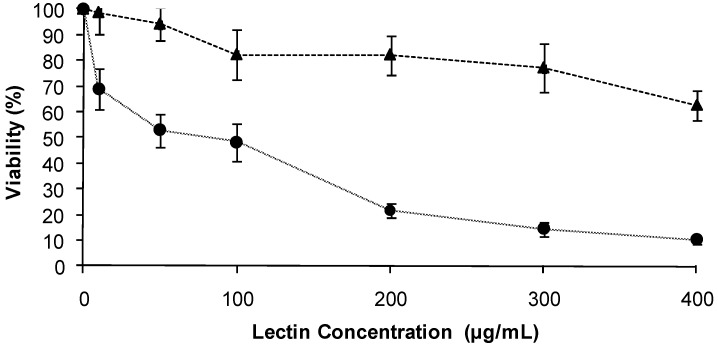
Effect of tepary bean lectins on the viability of human malignant cells lines. Cells (C33-A, SW480) were exposed to the indicated concentration of tepary bean lectins for 24 h. The viability of cells was determinate as described in the Experimental section. Results are presented as the percentage of viable cells (untreated cells was considered as 100% of viability). C33-A 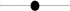 SW480 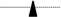.

The initial experiments were conducted employing the MTT assay to examine the cytotoxic effects of TBL. The cytotoxicity effects of tepary bean lectin on Sw480 and C33-A cells are shown in Figure 2. The results of this experiment demonstrated that TBL inhibited significantly the growth of Sw480 and C33-A cells lines. The IC_50_ of TBL for Sw480 cells was 84.2 µg/mL and more than 400 µg/mL for C33-A, indicating that Sw480 cells are more sensitive to the action of TBL. Thus, 0, 10, 25, 50 and 100 µg/mL of lectin concentration were chosen for the subsequent experimental assays.

### 2.3. Cytotoxic effect of TBL in human malignant cells

The effect of TBL in malignant cells was evaluated using a radiolabeled ^3^[H] thymidine assay. The data (Table 2) shows a dose dependent effect in both cell lines, so that increasing the concentration of lectin (TBL), decreased thymidine incorporation, suggesting a decrease in cell proliferation, although the effect is greater in C33-A cells lines, on the other hand, at 100 µg/mL shows an elevation in thymidine incorporation, which is associated with increased proliferative capacity of cells.

**Table 2:** Effect of tepary bean lectin on DNA synthesis in human malignant cells.

Treatment	DNA (% incorporation 3[H] thymidine)	
	SW480	C33-A
Control	100 ± 10.56	100 ± 11.06
10 µg/mL	71.3 ± 5.93	42.6 ± 11.35
25 µg/mL	66.7 ± 6.19	23.6 ± 12.08
50 µg/mL	59.7 ± 5.43	21.2 ± 13.47
100 µg/mL	33.4 ± 18.77	43.1 ± 4.96

Cells were exposed to the indicated concentration of lectins for 24 h, after with ^3^[H]-thymidine

### 2.4. Colony formation

**Figure 3:**
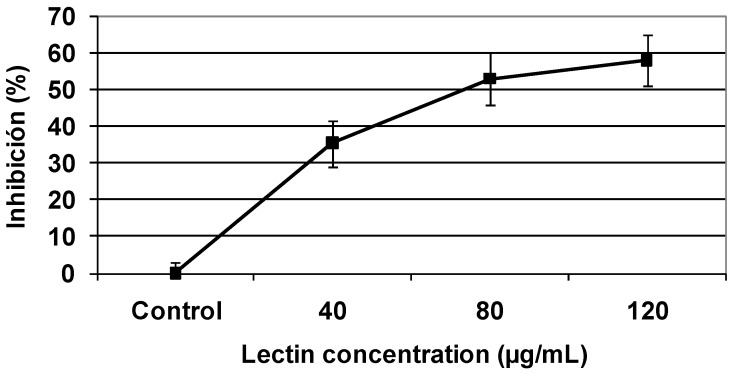
Inhibitory effect of tepary bean lectin on colony formation of the human colon cancer cell line (Sw480). Cells were exposed to the indicated concentration of lectin for 1 h. Then the number of cells that were able to form colonies was determined as described in the Experimental section. Results are presented as the percentage of the inhibition of colony formation. Each value represents the mean (± SD) obtained from triplicate plates.

**Figure 4:**
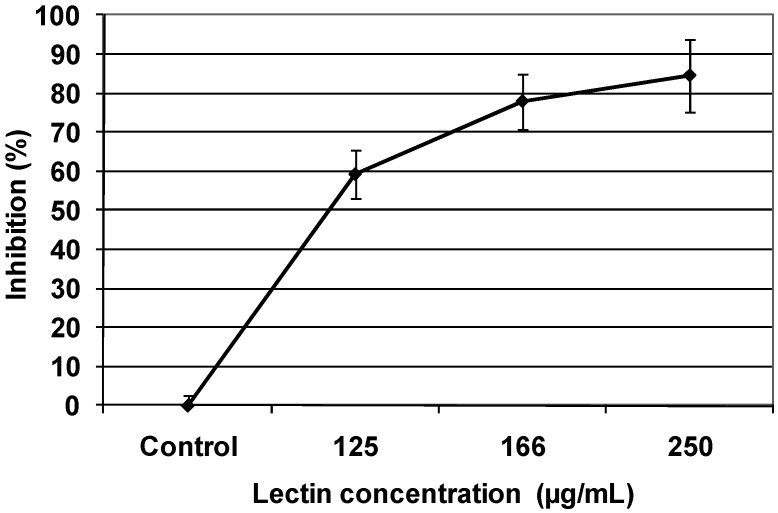
Inhibitory effect of tepary bean lectin on colony formation of the human epithelial cervical carcinoma cell line (C33-A). Cells were exposed to the indicated concentration of lectin for 1 h, then the number of cells that were able to form colonies was determined as described in the Experimental section. Results are presented as the percentage of the inhibition of colony formation. Each value represents the mean (± SD) obtained from triplicate plates.

Colony formation assays were carried out to examine the effect of TBL on the ability to form colonies. Sw480 and C33-A cells were incubated with TBL at various concentrations for 1 h, as indicated in Figure 3 and Figure 4. In these figures, a dose dependent inhibitory effect on colony formation in both cells lines can observed, being Sw480 cells lines more sensitive (84% inhibition) than C33-A cells (58% inhibition).

### 2.5. Discussion

Today the main problem in the use of drugs in cancer treatment is the potential toxicity of these agents to normal cells; for this reason chemoprevention employing natural agents for the treatment and prevention cancer is a good and new alternative. For this reason since several years ago, studies that analyze the anticarcinogenic activity of many plants extracts or pure phytochemical compounds have been reported [31,32]. Some of these studies, performed *in vitro* and *in vivo*, have shown that phytochemical compounds have the capacity to inhibit the viability of malignant cells as well as the growth of tumors in animals [33,34]. Some of these compounds are proteins such as lectins, and there is plenty of evidence that pure lectins, or protein extracts enriched with these proteins possess cytotoxic effects in normal or malignant cells from different origins [19,35]. For this reason in the present work, tepary bean lectin was purified with the aim of studying its anticarcinogenic potential on human malignant cells lines such as of colon cancer (Sw480) and cervical cancer (C33-A).

Our results show that tepary bean lectin has cytotoxic effects on both malignant cells lines, evidenced by a decrease in the cellular viability of approximately 30% and 90%, respectively (Figure 2); as well as an inhibitory effect on DNA synthesis, shown by a decrease in ^3^[H]-tymidine incorporation to 67% for Sw480 cells and 57% for C33-A cells after 24 hours.

Colony formation assays confirmed the TBL effect on the proliferation of both cells lines; being this effect higher on Sw480 cells (98%) than in C33-A cells (59%). This effect increased over several cell cycles. Many reports have suggested that the inhibition of nucleic acid synthesis in tumor cells by lectins is a likely cause for their cytotoxic effect on normal as well in malignant cells [8,9].

The differences observed by this study on the effect of tepary bean lectin on the viability and proliferation of Sw480 and C33-A cells can be explained by variations in the carbohydrate composition on the membrane surface between these cell lines [36,37].

Many reports have demonstrated that plant lectins have other effects in tumor cell recognition, cell adhesion and localization, signal transduction across membranes, mitogenic stimulation, apoptosis and citotoxicity [15,26]. Other research groups have demonstrated differences in the binding pattern of lectins to malignant cells [10]. Additionally, the anticancer effect of lectins has been documented [26,27,38,39]. Lectins have different toxic capabilities on malignant and normal cells and this differences can be explained due to the presence of different carbohydrate structures on the surface membranes among these cells [15,40].

## 3. Experimental

### 3.1. Extraction and purification of lectins

Tepary bean (*Phaseolus acutifolius*) seeds were purchased in a local market in Hermosillo (Sonora, México). The beans were ground in a Wiley mill fitted with a 60 mesh screen. The tepary lectin was extracted as described by González de Mejía *et al.* [41]. The flour was extracted during 16 h at 4 °C with 10 mM phosphate buffered saline PBS solution at a 1:10 (w/v) flour:buffer ratio. The mixture was centrifuged at 12,000 g for 30 min to remove the insoluble residues; the supernatant was then subjected to a protein precipitation with 70% (NH_4_)_2_SO_4_. After centrifugation at 12,000 g, the precipitate was resuspended and dialyzed overnight against PBS buffer and stored at −20 °C. The dialysis fraction was subjected to affinity chromatography in a FPLC system (Amersham Biosciences) fitted with a fetuin-agarose column. After injection, the unbound material was eluted with 20 volumes of PBS and the bound protein fraction was eluted from the column with 20 volumes of 50 mM glycine-HCl, pH 2.5. The fractions that were active in the hemagglutination assay (see below), were pooled and dialyzed against distilled water at 4°C and subsequently freeze-dried.

### 3.2. Quantification of protein

The protein concentration was determined by the method of Lowry *et al.* [42] using bovine serum albumin as standard. 

### 3.3. Hemagglutination assays

The hemagglutination activity assay was carried out in 96 well microtiter plates. The fetuin bound lectin was diluted serially (2-fold), adjusting the sample volume in each well to 50 µL with PBS. Diluted samples were mixed each with 50 µL of the 2% suspension of trypsinized human erythrocytes type A and O. The reaction mixtures were incubated 1 h at room temperature and then observed visually for positive agglutination. The titer was defined as the reciprocal of the highest dilution showing detectable agglutination [43].

### 3.4. Polyacrilamide gel electrophoresis (PAGE)

The purified lectin was subjected to electrophoretic analysis by native PAGE under alkaline conditions in an 11% gel according to the method of Laemmli [44]. After being loaded into the gel, the protein samples were electrophoresed at a constant current of 20 mA at 4 °C. For dissociation of the protein subunits, sodium dodecyl sulfate (SDS) PAGE was carried out according to the method by Laemmli [44] under reducing conditions (with β-mercaptoethanol) in a 12% gel. Native and reducing SDS PAGE runs were performed in a vertical mini protean II electrophoretic system (BioRad). Separated proteins in the gels were visualized by staining with Coomasie brilliant blue R-250.

### 3.5. Cell culture

Cytotoxicity assays described bellow were carried out using two human malignant cells lines, Sw480 (human, Caucasian, colon, adenocarcinoma) and C33-A (human epithelial cervical carcinoma). These cell lines were obtained from the American Type Culture Collection (ATCC, Rockville, MD, USA). The cells were grown in Dulbecco´s modified Eagle´s medium (DMEM) (Gibco, Grand Island, NY, USA), supplemented with 10% fetal bovine serum (FBS, Gibco) in a CO_2_ water jacketed incubator (Nuaire, Plymouth, MN, USA) at 37 °C in humidified atmosphere consisting of 5% CO_2_ and 95% air.

### 3.6. Cell viability assay

The tetrazolium dye colorimetric test using (3-(4, 5-dimethylthiazol-2-yl)-2-5-diphenyltetrazolium bromide (MTT) was used to determinate the viability of Sw480 and C33-A cells lines. The MTT assay is based on the ability on functional mitochondria to catalyze the reduction of (3-(4, 5-dimethylthiazol-2-yl)-2-5-diphenyltetrazolium bromide to an insoluble formazan product, the concentration of which can be measured spectrophotometrically [45]. Sw480 and C33-A cells lines were first cultured in 96 well microplates (1.0 × 10^4^ cells/well) in DMEM for 24 h. After incubation, cells were washed twice with PBS, and pretreated with different concentration (0 to 400 μg/mL) of TBL. After 24 hours of incubation with the lectin solution, the cells were washed with PBS and each well was added with MTT reagent (5 mg/mL), and the plate was incubated at 37 °C for an additional 3 h. After this cuncubation, media was removed, and the intracellular formazan product was dissolved in 100 μL dimethyl sulfoxide (DMSO). The absorbance of each well was then measured at 540 nm, and the percentage of cell viability was calculated.

### 3.7. Cytotoxicity assay

For the cytotoxicity assays, the cells were cultured in flat bottomed plates at a concentration of 5 × 10^4^ cells/well and incubated with different TBL concentrations (0 to 100 µg/mL) in DMEM for 24 h. The cell proliferation was measured by detecting the incorporation of tritium labeled thymidine (3 µCi/well) during an incubation period of 30 min. To stop uptake of radioactivity, the medium was removed and the cells were washed twice with 10 mM PBS solution pH 7.6. The cells were then lysed by adding 500 µL of SDS 0.1% containing 10 mM EDTA (pH 7.4) to each well. After 30 min of incubation at room temperature, the lysate was added with 500 µL of 10% cold trichloroacetic acid (TCA) and the precipitate was collected in a nitrocellulose paper, washed three times with TCA 5%, dried, added with scintillation cocktail (4 mL) and counted in a liquid scintillation counter (Beckman LS 65000). All determinations were done in triplicate.

### 3.8. Colony formation assay

To examine the effect of TBL on tumor cell colony formation, Sw480 and C33-A cells lines were cultured in 100 mm Petri dishes at 2 × 10^4^ cells per dish with 10 mL DMEM medium. After two days, different concentration of tepary bean lectins (C33-A: 0 to 120 µg/mL; Sw480: 0 to 250 µg/mL) were added and incubation continued for 1 h, cultures were the trypsinized (0.05 M trypsin), counted and 200 cell were seeded in 60 mm Petri dishes for determination of colony formation. At least three replicate colony determinations were made for each culture. After 10 days of incubation, the resulting colonies were rinsed with 50 mM phosphate buffer, pH 7.6 containing 150 mM NaCl, fixed with methanol, stained with Giemsa (Sigma) and the number of colonies (with diameter > 0.05 mm) per dish were determined as described previously [46]. Inhibition is defined as the ratio of the colony numbers in the treated group to that in the control group.

## 4. Conclusions

In conclusion, the present study has shown that tepary bean (*Phaseolus acutifolius*) lectin has the ability to inhibit the growth of cancer cells, either by causing cytotoxic or anti-proliferative effects on both the cells lines investigated, and by inhibiting colony formation, being the effects of the lectin more pronounced on Sw480 cells than in C33-A cells. It is known that lectins recognize carbohydrates, and in previous studies with cells, it as come to be known that lectins interact with the carbohydrates present in the external membrane of the cells, and that this bond forms a covalent link; thus, the bond causes cellular modifications, affecting metabolism, cellular division, etc. On the other hand, this lectin could be a candidate to study further for its use in the treatment and prevention of cancer, with different normal and cancerous cell lines, *in vivo* studies, toxicity studies, etc. 
